# Green Tea Polyphenols Attenuated Glutamate Excitotoxicity via Antioxidative and Antiapoptotic Pathway in the Primary Cultured Cortical Neurons

**DOI:** 10.1155/2016/2050435

**Published:** 2015-12-15

**Authors:** Lin Cong, Chang Cao, Yong Cheng, Xiao-Yan Qin

**Affiliations:** ^1^Beijing Engineering Research Center of Food Environment and Health, Minzu University of China, Beijing 100081, China; ^2^Department of General Surgery, Peking Union Medical College Hospital, Chinese Academy of Medical Sciences and Peking Union Medical College, Beijing 100730, China; ^3^College of Life & Environmental Science, Minzu University of China, Beijing 100081, China; ^4^Laboratory of Neurobiology and State Key Laboratory of Biomembrane and Membrane Biotechnology, College of Life Sciences, Peking University, Beijing 100871, China

## Abstract

Green tea polyphenols are a natural product which has antioxidative and antiapoptotic effects. It has been shown that glutamate excitotoxicity induced oxidative stress is linked to neurodegenerative diseases such as Alzheimer's disease and Parkinson's disease. In this study we explored the neuroprotective effect of green teen polyphenols against glutamate excitotoxicity in the primary cultured cortical neurons. We found that green tea polyphenols protected against glutamate induced neurotoxicity in the cortical neurons as measured by MTT and TUNEL assays. Green tea polyphenols were then showed to inhibit the glutamate induced ROS release and SOD activity reduction in the neurons. Furthermore, our results demonstrated that green tea polyphenols restored the dysfunction of mitochondrial pro- or antiapoptotic proteins Bax, Bcl-2, and caspase-3 caused by glutamate. Interestingly, the neuroprotective effect of green tea polyphenols was abrogated when the neurons were incubated with siBcl-2. Taken together, these results demonstrated that green tea polyphenols protected against glutamate excitotoxicity through antioxidative and antiapoptotic pathways.

## 1. Introduction

Glutamate is a major neurotransmitter in the mammalian central nervous system. Glutamatergic neurons form the main excitatory system in the brain and play a pivotal role in many neurophysiological functions [1]. However, excessive release of glutamate can lead to neuronal dysfunction and cell death in a process now referred to as excitotoxicity [2]. Remarkably, glutamate excitotoxicity had been shown to play an important role in many neurodegenerative diseases including Alzheimer's disease (AD), Parkinson's disease (PD), and Huntington's disease (HD) [3]. Growing evidence suggests that the mitochondrial dysfunction mediated by glutamate excitotoxicity-induced oxidative stress was associated with both acute and chronic neurodegenerative disorders [4]. Moreover, it has been shown that glutamate excitotoxicity was involved in the pathogenesis of inflammatory pain [5]. In fact, reactive oxygen species have also been implicated in the cascade of events resulting from inflammatory pain [5, 6]. Since glutamate excitotoxicity and oxidative stress are two common features for various neurodegenerative diseases [3] and inflammatory pain, searching for drugs or natural products targeting glutamate excitotoxicity and oxidative stress is a good strategy for potential treatments of neurodegenerative diseases and inflammatory pain.

Recently, a rapidly growing number of polyphenolic compounds with neuroprotective effects have been described [7]. The most discussed effects of polyphenols are the antioxidant properties. Dietary intake of polyphenols is known to attenuate oxidative stress and reduce the risk for related neurodegenerative diseases such as AD, PD, and HD [8]. Interestingly, green tea polyphenols have been found to be more antioxidant active than vitamin C and vitamin E [9]. Furthermore, green tea polyphenols were shown to protect against beta-amyloid toxicity in the primary cultured cortical neurons [10]. Further study confirmed the potential beneficial effect of green tea polyphenols in AD as green tea polyphenols demonstrated inhibition of acetylcholinesterase [11]. However, the effects of green tea polyphenols on excitotoxicity are still poorly understood.

In this study we have explored the potential protection of green tea polyphenols against glutamate excitotoxicity. We found that glutamate induced neurotoxicity in the primary cultured cortical neurons was inhibited by green tea polyphenols. We then showed that green tea polyphenols rescued glutamate induced oxidative stress. We further demonstrated that green tea polyphenols restored the dysfunction of pro- and/or antiapoptotic proteins Bax, caspase-3, and Bcl-2 caused by glutamate excitotoxicity. More interestingly, we used SiBcl-2 and showed that the neuroprotection of green tea polyphenols was abrogated with the treatment of SiBcl-2 in the neurons. Thus our results demonstrated that green tea polyphenols attenuated glutamate excitotoxicity via antioxidative stress and antiapoptotic pathway.

## 2. Materials and Methods

### 2.1. Primary Cortical Neuron Culture

Primary cortical neuron cultures were prepared as described previously [12]. Briefly, cortical tissues were dissected and dissociated. Then the cells were centrifuged and resuspended in DMEM with 10% fetal bovine serum (Invitrogen, CA, USA), 2 g/L HEPES (Invitrogen), 100 *μ*g/mL penicillin, and 100 *μ*g/mL streptomycin (Invitrogen). Cells were then plated in poly-L-lysine-coated (Sigma, St. Louis, Missouri) plates or coverslips. Each well was plated with 1 × 10^6^ cells for 12-well plates, 5 × 10^5^ cells for 24-well plates, and 1 × 10^5^ cells for 96-well plates. The cultured neurons were maintained in an incubator with 5% CO_2_ and 95% O_2_ at 37°C and were treated at 5–7 days in culture. 10 *µ*M cytosine arabinoside (Sigma) was added to the medium to inhibit the growth of glial cells after three days of culture.

### 2.2. Reagents and Treatments

The property of green tea polyphenols with 98% purity has been described previously [10]. Green tea polyphenols were added freshly into culture medium during treatments for 24 h; then glutamate was added to the culture medium for 24 h to induce excitotoxicity in the primary cultured cortical neurons. In some experiments, the neurons were transiently transfected with 20 nM siRNA oligonucleotides directed against Bcl-2 (SiBcl-2) or a scrambled control sequence (SiScr) (Invitrogen) for 24 h using Lipofectamine RNAiMAX (Invitrogen) according to the manufacturer's instruction.

### 2.3. Cell Viability and Death Assays

The cell viability was tested as described previously [13]. This was achieved by the neurons' ability to reduce the dye methyl thiazolyl tetrazolium (MTT, Sigma, MO) to its insoluble formazan, which has a purple color. Briefly, MTT was added to the 96-well plates after various treatments in the primary cultured cortical neurons and the plate was maintained in an incubator for 2 h. Then the supernatants were discarded and 150 *µ*L DMSO was added to each well to dissolve the formazan crystal. The absorption was recorded at 570 nm using a Bio-Rad plate reader (Thermo, MA, USA).

Cell death was measured by TUNEL assay as described previously [14]. Briefly, the primary cultured cortical neurons were fixed by 4% paraformaldehyde after treatments. Then the cells were permeabilized by 0.1% Triton X-100 with 0.1% sodium citrate on ice for 2 minutes. After that,* in situ* cell death detection kit was used for terminal deoxynucleotidyl transferase mediated dUTP nick-end labeling (TUNEL) staining to detect apoptotic cells as described by the manufacturer (Roche, IN, USA).

### 2.4. ROS and SOD Measurements

Reactive oxygen species (ROS) release in the primary cultured cortical neurons was measured by Cellular Reactive Oxygen Species Detection Assay Kit (Abcam). It used the cell permeant reagent 2′,7′-dichlorofluorescin diacetate (DCFDA); DCFDA is deacetylated by cellular esterases after diffused into the cells, which is later oxidized by ROS into 2′,7′-dichlorofluorescein (DCF). DCF is a highly fluorescent compound which can be detected by fluorescence spectroscopy. The experiments were performed according to the manufacturer's instruction.

Superoxide Dismutase (SOD) activity was assessed by measuring the dismutation of superoxide radicals generated by xanthine oxidase and hypoxanthine. The measurement of SOD activity in the neurons was achieved by Superoxide Dismutase Assay Kit (Cayman Chemical) as described by the manufacturer.

### 2.5. Western Blots

Western blots were used to test the protein levels after various treatments in the primary culture cortical neurons as described previously [15]. Briefly, the protein lysates were harvested from the neurons. Denatured protein samples with equal amounts were separated and transferred to PVDF membrane (Millipore, MA, USA). After blocking, the membranes were probed with primary antibodies followed by secondary antibodies. Protein bands were then detected by Bio-Rad ChemiDoc system (Bio-Rad, CA, USA) with the presence of enhanced chemiluminescence reagents. The following primary antibodies were used in the experiments: purified polyclonal rabbit anti-*β*-actin antibody (Santa Cruz, CA, USA), monoclonal rabbit anti-activated caspase-3 antibody (Cell Signaling, MA, USA), polyclonal rabbit anti-Bcl-2 antibody (Santa Cruz), and polyclonal rabbit anti-Bax antibody (Santa Cruz).

### 2.6. Statistical Evaluation

All data in the experiments are presented as means ± SEM. Statistical significance (^*∗*^  or ^#^ *p* < 0.05, ^*∗∗*^  or ^##^ *p* < 0.01, and ^*∗∗∗*^  or ^###^ *p* < 0.001) among groups was measured by one-way analysis of variance (ANOVA) followed by Tukey* post hoc* multiple comparisons tests.

## 3. Results

### 3.1. TP Attenuated Glutamate Excitotoxicity in Primary Cultured Cortical Neurons

**Figure 1:**
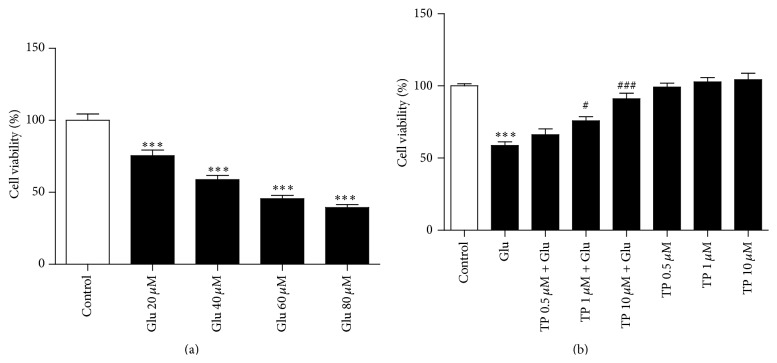
TP protected against glutamate (Glu) induced neurotoxicity in the primary cultured cortical neurons. (a) Glutamate reduced the cell viability at a dose dependent manner in the primary cultured cortical neurons as tested by MTT assay. (b) The glutamate induced reduction of cell viability was partially rescued by 1 *μ*M or 10 *μ*M TP in the neurons, while TP alone had no effect on the cell viability of the neurons. Glu: glutamate. At least three independent experiments were performed. Data shown represent one experiment. *n* = 5/group. One-way analysis of variance (ANOVA) followed by Tukey* post hoc* multiple comparisons tests, *F*_(4,20)_ = 55.25 for (a); *F*_(7,32)_ = 29.69 for (b). ^*∗∗∗*^ compared to the control group and ^#^  or ^###^ compared to the Glu group.

In order to confirm the glutamate excitotoxicity, we treated the primary cultured cortical neurons with 20 *μ*M, 40 *μ*M, 60 *μ*M, or 80 *μ*M glutamate for 24 h; then MTT assay was used to measure the cell viability. As shown in Figure 1(a), glutamate reduced the cell viability at a dose dependent manner in the primary cultured cortical neurons. We next tested whether green tea polyphenols (TP) could rescue glutamate induced neurotoxicity in the neurons and pretreated the primary cultured cortical neurons with vehicle, 0.5 *μ*M, 1 *μ*M, or 10 *μ*M for 24 h; then 40 *μ*M glutamate was added to the neurons for 24 h. Our results showed that pretreatment with TP reduced glutamate induced neurotoxicity at a dose dependent manner as tested by MTT assay (Figure 1(b)). However, TP alone had no influence of the cell viability in the neurons (Figure 1(b)). We used 10 *μ*M TP in the following experiments since it had the maximal neuroprotection.

**Figure 2:**
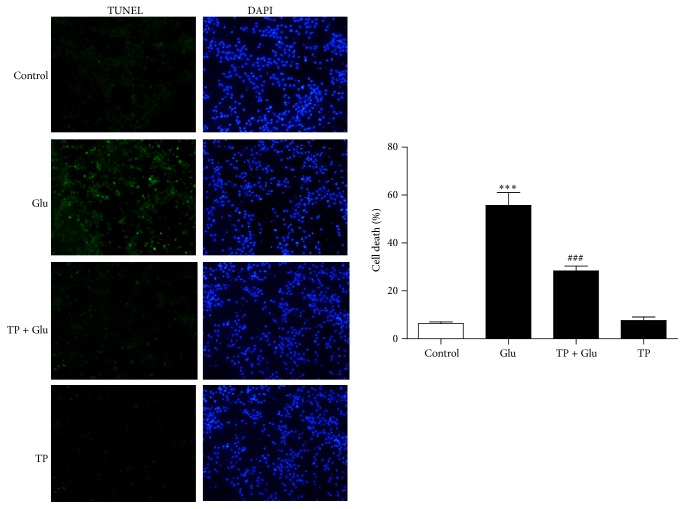
TP attenuated glutamate induced cell death in the neurons. Photomicrographs and histograms and show that TP inhibited glutamate induced apoptosis as assessed by TUNEL assay and there is no marked influence of TP alone on the number of died cells in the neurons. At least three independent experiments were performed. Data shown represent one experiment. *n* = 3/group. One-way analysis of variance (ANOVA) followed by Tukey* post hoc* multiple comparisons tests, *F*_(3,8)_ = 58.52. ^*∗∗∗*^ compared to the control group. ^###^ compared to the Glu group.

To confirm the neuroprotective effect of TP against glutamate excitotoxicity, we used TUNEL assay to measure the apoptosis. Our results showed that 40 *μ*M glutamate significantly increased the number of apoptotic cells in the primary culture cortical neurons, while pretreatment of the neurons with TP significantly reduced the cell death induced by glutamate (Figure 2). The above results demonstrated that TP protected against glutamate induced neurotoxicity in the primary culture cortical neurons.

### 3.2. TP Inhibited Glutamate Induced Oxidative Stress in the Neurons

**Figure 3:**
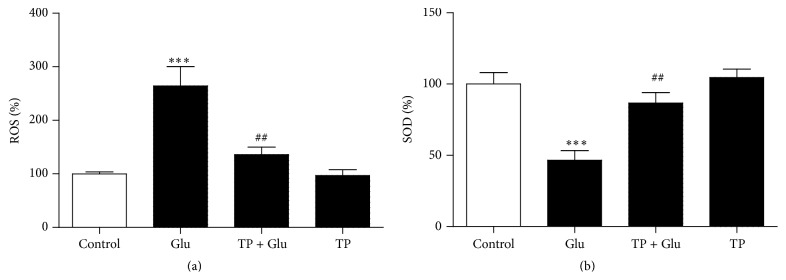
TP reduced oxidative stress induced by glutamate in the neurons. (a) Bar graphs show that glutamate induced ROS release in the primary cultured cortical neurons was prevented by incubation of TP. (b) Bar graphs show that the SOD activity in the neurons was downregulated by the glutamate, while TP treatment rescued the SOD activity. Two independent experiments were performed. Data shown represent one experiment. *n* = 5/group. One-way analysis of variance (ANOVA) followed by Tukey* post hoc* multiple comparisons tests, *F*_(3,16)_ = 14.97 for (a); *F*_(3,16)_ = 13.92 for (b). ^*∗∗∗*^ compared to the control group and ^##^ compared to the Glu group.

We next explored the mechanism by which TP protected against glutamate excitotoxicity. We measured ROS release since it contributes to glutamate induced neuronal death. Our results confirmed that glutamate caused the production of ROS in the primary cultured cortical neurons; we further showed the pretreatment of TP inhibited the production of ROS caused by glutamate (Figure 3(a)). Moreover, we found that TP rescued the loss of SOD activity induced by glutamate in the primary cultured cortical neurons (Figure 3(b)). Thus our data demonstrated the glutamate induced oxidative stress in the neurons was rescued by TP.

### 3.3. Bcl-2 Mediated the Neuroprotection of Glutamate in the Neurons

**Figure 4:**
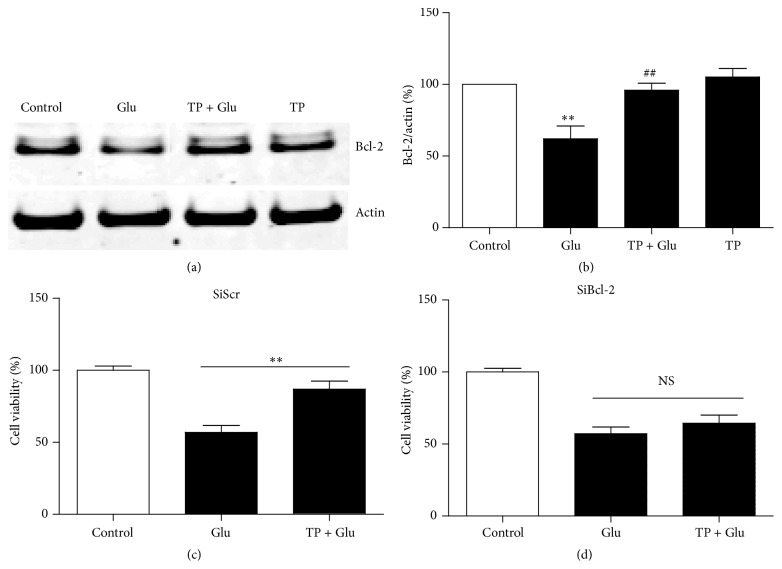
Involvement of Bcl-2 in the neuroprotection of TP. (a) Representative Western blot analysis of Bcl-2 protein in primary cultured cortical neurons after various treatments. Actin served as internal control. (b) Summary of the optical density of Bcl-2 normalized to actin. Results show that glutamate induced decrease of Bcl-2 was rescued by treatment of TP in the neurons. At least three independent experiments were performed. Data were from all the experiments combined. *n* = 4/group. One-way analysis of variance (ANOVA) followed by Tukey* post hoc* multiple comparisons tests, *F*_(3,12)_ = 11.18. ^*∗∗*^ compared to the control group and ^##^ compared to the Glu group. ((c), (d)) Bar graphs show that TP inhibited glutamate induced neurotoxicity in SiScr treated cortical neurons but not in SiBcl-2 treated neurons as tested by MTT assay. Two independent experiments were performed. Data shown represent one experiment. *n* = 5/group. One-way analysis of variance (ANOVA) followed by Tukey* post hoc* multiple comparisons tests, *F*_(2,12)_ = 23.19 for (c); *F*_(2,12)_ = 26.15 for (d).

The involvement of antiapoptotic protein Bcl-2 in the neuroprotection of TP against glutamate excitotoxicity in the primary cultured cortical neurons was further investigated. The cultured cortical neurons were treated with vehicle or 40 *μ*M glutamate. As shown in Figures 4(a) and 4(b), the content of Bcl-2 in the neurons decreased significantly after treatment of glutamate compared to the control group tested by Western blot. Furthermore, pretreatment with TP rescued the glutamate induced decrease in the content of Bcl-2 in the cultured neurons. To find out whether Bcl-2 mediated the neuroprotection of TP in the neurons, we used siBcl-2. As shown in Figure 4(c), TP attenuated glutamate induced neurotoxicity when the neurons were treated with SiScr. In contrast, the neuroprotective effect of TP against glutamate excitotoxicity was almost completely blocked when the neurons were treated with SiBcl-2 (Figure 4(d)). Thus our data indicated that the neuroprotection of TP against glutamate induced neurotoxicity was mediated by Bcl-2.

### 3.4. Effects of TP on the Glutamate Induced Dysregulation of Bax and Caspase-3

**Figure 5:**
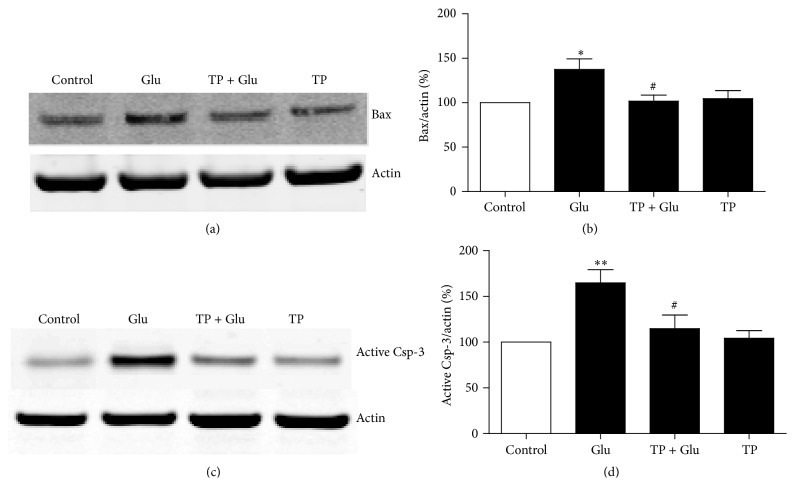
Influence of TP on glutamate induced dysregulation of Bax and caspase-3 (Csp-3). (a) Representative Western blot analysis of Bax expression in primary cultured cortical neurons after various treatments. Actin served as internal control. (b) Summary of the optical density of Bax normalized to actin. Results show that TP inhibited glutamate induced upregulation of Bax in the neurons. (c) Representative Western blot analysis of active casapse-3 in primary cultured cortical neurons after various treatments. Actin served as internal control. (d) Summary of the optical density of active caspase-3 normalized to actin. Results show that TP inhibited glutamate induced activation of caspase-3 in the neurons. At least three independent experiments were performed. Data were from all the experiments combined. *n* = 4/group. One-way analysis of variance (ANOVA) followed by Tukey* post hoc* multiple comparisons tests, *F*_(3,12)_ = 4.533 for (b); *F*_(3,12)_ = 7.162 for (d). ^*∗*^  or ^*∗∗*^ compared to the control group and ^#^ compared to the Glu group.

We also tested the proapoptotic proteins Bax and caspase-3 after various treatments. Our results showed that glutamate increased the expression of Bax in the primary cultured cortical neurons, while pretreatment with TP inhibited the glutamate induced upregulation of Bax in the neurons (Figures 5(a) and 5(b)). Moreover, we found that the glutamate induced activation of caspase-3 was inhibited by treatment of TP in the neurons (Figures 5(c) and 5(d)). The above results suggest the involvements of Bax and caspase-3 in the neuroprotection of TP against glutamate neurotoxicity in the primary cultured cortical neurons.

## 4. Discussion

Neurodegenerative diseases are a wide class of hereditary and sporadic conditions characterized by progressive nervous system dysfunction. These disorders include Alzheimer's disease (AD), Parkinson's disease (PD), Huntington's disease (HD), and amyotrophic lateral sclerosis (ALS) which are caused by a combination of genetic and environmental factors. AD alone affects approximately 4.5 million Americans, projected to increase to 11 and to 16 million by 2050 [16]. With extensive research and clinical trials globally, neurodegenerative diseases remain without curative treatments, and the available therapies provide only symptom improvements. In AD, various neurotrophic factors such as brain-derived neurotrophic factor (BDNF), fibroblast growth factor 2 (FGF2), neurotrophic factor-*α*1 (NF-*α*1), and activity-dependent neuroprotective protein (ADNP) have been studied as potential therapeutic targets for treating the malicious disease [17–20], yet there are no successful drugs developed based on these trophic factors. Thus looking for new neuroprotectants is needed for control of neurodegenerative diseases. The most suitable approaches to dissect the factors and fully study the cellular and molecular mechanisms of these diseases are global “omics” approaches. In fact, a lot of information has been gained from genomic profiling of cortical neurons following exposure to beta-amyloid [21]. Furthermore, proteomics approaches are the most innovative tools for monitoring at the proteome level the extent of protein oxidative insult and related modifications with the identification of targeted proteins [22]. Natural products such as polyphenols have now been emerging as new therapeutic targets for treating neurodegenerative diseases. In our study, we demonstrated that glutamate induced neurotoxicity was attenuated by green tea polyphenols in the primary cultured cortical neurons; our data are consistent with the previous findings of the antioxidant effect of green tea polyphenols.

Glutamate induced neuronal death is associated with the production of ROS in the neurons [23]. In this study we showed that glutamate induced ROS release in the primary cultured cortical neurons which is consistent with previous literature. Pretreatment with green tea polyphenols prevented the excessive release of ROS caused by glutamate in the neurons, confirming the neuroprotection of green tea polyphenols. We further demonstrated that the activity of the antioxidant enzyme SOD was downregulated by glutamate excitotoxicity and green tea polyphenols rescued the activity of SOD. These results suggest that the antioxidant role of green tea polyphenols might play a critical role against glutamate induced neurotoxicity.

The mitochondria have been identified as a major source of ROS, and their dysfunction with time appears to contribute to neural decay and aging [24]. Mitochondria play a key role in the regulation of apoptotic cell death and the ratio of proapoptotic to antiapoptotic Bcl-2 family members is a major checkpoint in the regulation of apoptosis [25]. The conformation of Bax is changed upon apoptosis signal; it is then translocated into the mitochondrial membranes from cytosol to form pores, which leads to the release of cytochrome c and subsequently activation of caspase to initiate cell death; however, the antiapoptotic protein Bcl-2 inhibits the mitochondria pore formation to prevent cell death [26]. Our data showed that glutamate downregulated the expression of Bcl-2 and green tea polyphenols rescued the expression Bcl-2. Furthermore, knockdown Bcl-2 by siRNA in the neurons blocked the neuroprotective property of green polyphenols against glutamate toxicity. Interestingly, it has been reported that cellular ROS levels can be decreased by the antiapoptotic protein Bcl-2, while lower ROS levels are observed in Bcl-2-expressing cells [27]. These results support the neuroprotective effect of green tea polyphenols against excitotoxicity and suggest that the recovery of mitochondria energetics may meditate the neuroprotection. We also showed that green tea polyphenols prevented the activation of caspase-3 induced by glutamate in the neurons. This observation is consistent with caspase being downstream target of mitochondrial apoptosis.

In conclusion, we have demonstrated that green tea polyphenols protected against glutamate induced excitotoxicity in the primary cultured cortical neurons. Furthermore, we showed that the protective effect of green tea polyphenols was mediated by attenuation of oxidative stress and antimitochondrial apoptotic pathway. Thus our study supports the hypothesis that polyphenols are potential therapeutic targets for treating neurodegenerative disease; further investigations into the function of polyphenols are justified.
